# *Spinacia oleracea* Modulates Radiation-Induced Biochemical Changes in Mice Testis

**DOI:** 10.4103/0250-474X.42980

**Published:** 2008

**Authors:** Rashmi Sisodia, Ritu K. Yadav, K. V. Sharma, A. L. Bhatia

**Affiliations:** Radiation Biology Laboratory, Department of Zoology, University of Rajasthan, Jaipur-302 004, India

**Keywords:** *Spinacia oleracea*, antioxidant, testis, radioprotection

## Abstract

The present study is an attempt to investigate the radioprotective efficacy of spinach against radiation induced oxidative stress, since its leaves are rich in antioxidants like carotenoids (β-carotene, lutein and zeaxanthin) and high content of proteins, minerals, vitamin C. For the experimental study, healthy Swiss mice were selected from an inbred colony and divided into four groups. Group I (normal) it did not receive any treatment. Group II (drug treated) was orally supplemented with extract of spinach extract once daily at the dose of 1100 mg/kg for fifteen consecutive days. Group III (control) received distilled water orally equivalent to spinach extract for fifteen days than exposed to 5 Gy of gamma radiation. Group IV (experimental) was also administered orally with spinach extract for 15 consecutive days once daily. Thereafter, exposed to single dose of 5Gy of gamma radiation. After the exposure mice were than sacrificed at different autopsy intervals viz. 1, 3, 7, 15 and 30 days. Testis was removed for various biochemical estimations viz. LPO, protein, cholesterol and glycogen. Radiation induced augmentation in lipid peroxidation, glycogen and cholesterol values were significantly ameliorated by supplementation of SE extract, whereas radiation induced deficit in protein content could be elevated. This indicates that spinach extract pre - treatment renders protection against various biochemical changes in the mice testis to some extent if taken continuously which might be due to synergistic effect of antioxidant constituents present in the spinach.

India has a rich heritage of medicinal plants, many of which have been explored for the various bioactivities since ages. However, the radio protective potential of plants have been hardly explored, though a large number of compounds from various plant sources have been shown to possess antioxidant properties^1^,^2^. There is clear evidence that consumption of fruits and vegetables is beneficial to health. Much of the evidence supporting the protective role of fruits and vegetables comes from epidemiological literature. The nature of the protective effects of the specific nutrients found in fruits and vegetables, such as β-carotene, vitamin C and vitamin E is not yet clearly known. Recent studies with vitamin E indicate that high dose can slow the progression of Alzheimer's disease^3^. With few exceptions, however a single nutrient is not packaged into a single food and the combination of nutrients found in foods might have greater protective effects than each nutrient alone.

Present investigation has been undertaken to search out common nutritional plants, which may prove efficient antioxidant and could be easily recommended in nutritional dietary course for the population residing in areas where they are continuously exposed to radiation, as prophylactic agent relieving people from the psychological stress of taking tablets.

*Spinacia oleracea* (Spinach (Eng); *Palak* (Hindi)) is a common herb, native of South Asia. Spinach leaves are eaten as vegetable and is reported to be a good source of minerals, vitamin B-complex, vitamin K, ascorbic acid, carotene (β-carotene, lutein, zeaxanthin), protein content (2.0% per 100 g of edible protein) and flavonoids, all which have been shown to possess antioxidant properties^4^,^5^. Recently studies reported the presence of a series of water soluble powerful natural antioxidants in spinach leaves extract and their biological activities were described^6^–^9^.

Studies have shown that dietary supplementation of spinach improves learning and memory in mice against radiation induced oxidative stress^10^. Bickford^11^ reported that spinach improves cerebellar physiology and motor learning in aged rats. It has been assumed that nutritional intervention to increased intake of phytoantioxidants may reduce the threat of free radicals. Testicular tissues are rich in polyunsaturated fatty acid content and poor in antioxidant defense; therefore, prone to attack by reactive oxygen species (ROS), which are capable of oxidation of proteins lipids and DNA leading to cellular damage. Hence, this work focuses on *Spinacia oleracea*, a high phytoantioxidant and its effect on mice testis against deleterious effect of radiation.

## MATERIALS AND METHODS

Adult male Swiss mice (6-8 weeks old, weighing 23±2 g) were selected from an inbred colony maintained in an air-cooled laboratory and housed under condition free of pathogens. These were maintained on standard mice feed (Hindustan Lever Ltd. Mumbai) and water was provided ad libitum at constant temperature (22±1.5°) and light (10 h L and 14 h D). Institutional Animal Ethical Committee (IAEC) approved this study.

The cobalt teletherapy unit (ATC-C9) at Cancer Treatment Center, Department of Radiotherapy, SMS Medical College and Hospital, Jaipur was used for irradiation. Unanaesthestized mice restrained in well-ventilated plastic boxes were exposed to whole-body gamma radiation (5 Gy) at a distance of 77.5 cm from the source (SSD) to deliver the dose rate of 1.072 Gy/m. The dose was calculated as per the physical decay table for ^60^Co.

### Preparation of plant extract and dose selection:

Fresh spinach leaves (*Spinacia oleracea* Linn. RUBHL No. 19867* family-Chenopodiaceae) (*Rajasthan University Botany Herbarium Library, where the plant was identified by a taxonomist in consultation with experts and given the plant number) were collected locally and air-dried and then powdered and extracted with methanol by refluxing for 48 h (16 h × 3 d) at 40°. Half a kilogram of spinach yields about 100 g powder when dried and this dried powder yields 20 g crude methanol extract, which was dissolved in the double distilled water (DDW) and the required concentration was prepared just before oral administration. Dose selection of *Spinacia oleracea* was done on the basis of drug tolerance study in our laboratory^7^. Different doses of *Spinacia oleracea* (200, 400, 600, 800, 1100, 1400mg/kg) were tested against gamma irradiation (9 Gy) and 1100 mg/kg per day was used as optimum dose for further experimentation.

### Experimental design:

Mice selected from an inbred colony were divided into 4 groups comprising 6 animals in each group; Group I (normal) mice did not receive any treatment, Group II (drug, SE) mice of this group were orally administered spinach extract (SE) dissolved in double distilled water for15 consecutive days once daily, Group III (control (IR)) mice received distilled water orally equivalent to spinach extract for 15 days, and then exposed to single dose of 5 Gy of gamma radiation and Group IV (experimental (SE+IR)) here mice received spinach extract which was orally administered, once daily for 15 consecutive days. One hour after final administration of last dose of spinach extract, mice were whole body exposed to single dose of 5 Gy gamma radiations as in group third. Treatment in all the groups was carried out at 10 A.M. Mice from all the groups have been autopsied at the same time (10 A.M) and intervals i.e. 1, 3, 7, 15 and 30 days post-irradiation. Testes were removed and homogenate was prepared and estimated for various biochemical changes viz. protein, glycogen, cholesterol and lipid peroxidation.

### Biochemical determinations:

Lipid peroxidation was measured using thiobarbituric acid reactive substance (TBARS) according to the method of Ohkhawa *et al*^12^. The testes were removed and immediately placed in cold 0.9% NaCl and washed in the same. Homogenate was prepared (1 g of tissue in 9 ml of 1.15 KCl) and 0.2 ml of the sample was taken for the assay. Three repeats of the assay from each animal were carried out. The absorbance was read at 532 nm.

Estimation of protein was based on the method reported by Bradford^13^. Ten percent homogenate was prepared (1 g of tissue in 9 ml of NaCl) and 0.1 ml of the sample was taken for the assay. Three repeats of the assay from each animal were carried out. The absorbance was read at 595 nm. Total cholesterol was estimated using Burchard method^14^. 10% homogenate was prepared (1 g of tissue in 9 ml of acetic acid) and 0.5 ml of the sample was taken for the assay. Three repeats of the assay from each animal were carried out. The absorbance was read at 550 nm against blank.

Estimation of glycogen was based on the method reported by Montgomery^15^. Homogenate was prepared by adding 100 mg tissue in 2 ml of 30% KOH and boiled for 2-3 min till the tissue was digested. Three repeats of the assay from each animal were carried out. The absorbance was read at 624 nm against blank.

### Statistical analysis:

The values were expressed as mean±SD. The difference between various groups was analyzed by student's *t*-test and ANOVA test. Significance level was set at p<0.01.

## RESULTS

### Lipidperoxidation (LPO):

**Fig. 1:**
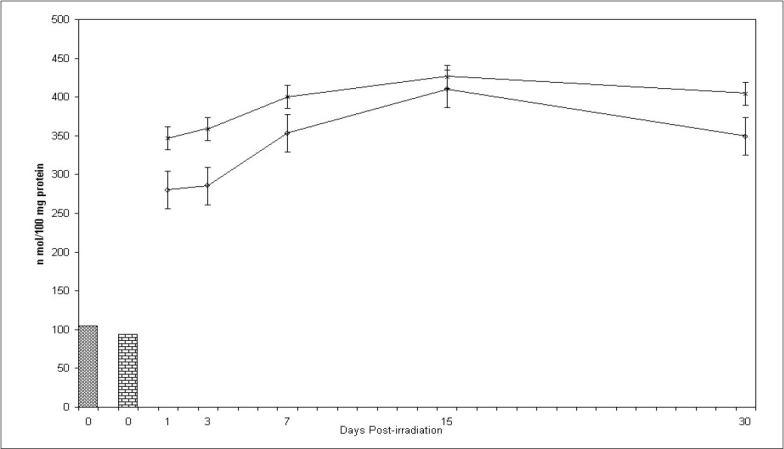
Lipid peroxidation (LPO) levels in mice testis at various post treatment days Variations in lipid peroxidation (LPO, TBARS nmol/100 mg protein±SD)) in testis of Swiss mice at various post treatment days, with and without supplementation of *Spinacia oleracea* extract (SE). Normal (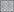) SE (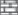), IR (–×–) and SE+IR (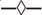)

Fig. 1 shows that LPO increases after irradiation in testis upto 15 days in both the groups experimental and control mice. Thereafter, decrease in LPO values was observed in both the groups indicating recovery. LPO values were significantly lower in experimental group from their respective control group at all the intervals noted. Percentage protection observed in LPO level in experimental group was 19.4, 20.5, 11.6, 12.4 and 13.7% at 1, 3, 7, 15 and 30 day post exposure, respectively. Difference between mean values of all four groups of lipid peroxidation was found to be significant (F=115.965, p<0.001).

### Protein:

**Fig. 2:**
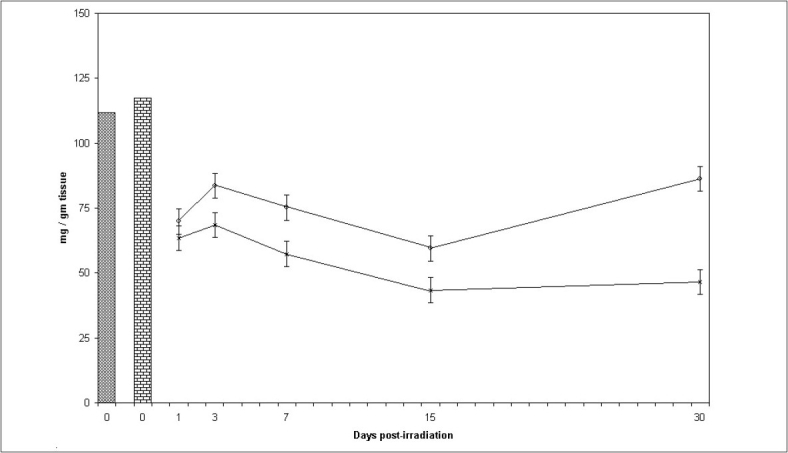
Protein levels in mice testis at various post treatment days Variations in protein level (mg/g tissue)+SD in testis of Swiss mice at various post treatment days, with and without supplementation of *Spinacia oleracea* extract (SE). Normal (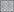) SE (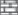), IR (––×––) and SE+IR (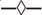)

There was continuous reduction in protein content upto day 15^th^ post irradiation except at day 3 where slight increase in protein concentration was recorded followed by an increase at day 30 in both groups (control and experimental). The values of protein content in the experimental group were significantly higher (p<0.001) than corresponding control mice at all the post irradiation days. Difference between mean values of all four groups of protein was highly significant. (F=74.654, p<0.000) (fig. 2).

### Glycogen:

**Fig. 3:**
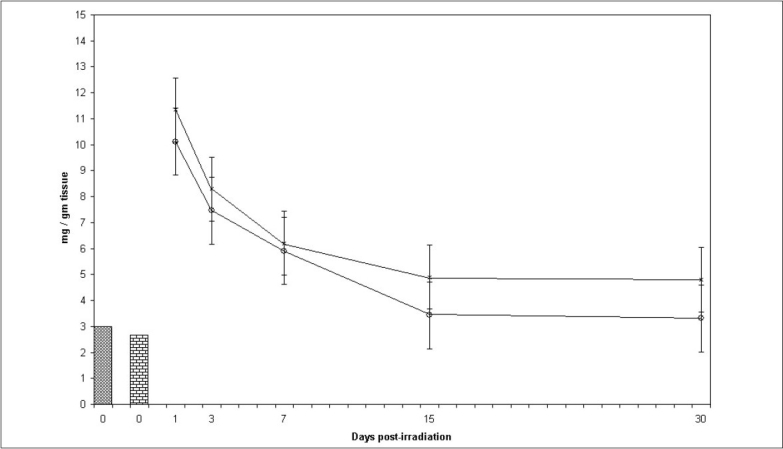
Glycogen levels in mice testis at various post treatment days Variations in glycogen levels (mg/g tissue)±SD in testis of Swiss mice at various post treatment days, with and without supplementation of *Spinacia oleracea* extract (SE). Normal (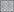) SE (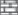), IR (––×––) and SE+IR (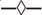)

A sharp increase in amount of glycogen by more than 3.79 and 3.09 times compared to the normal was observed in control and experimental groups respectively at 24hrs, after irradiation (fig. 3). Thereafter, it decreased continuously till the last interval studied in both the groups. Total glycogen levels were lowered in the experimental group after diet supplementation with spinach. Difference between mean values of all four groups of glycogen was significant (F= 6.197, p<0.005).

### Cholesterol:

**Fig. 4:**
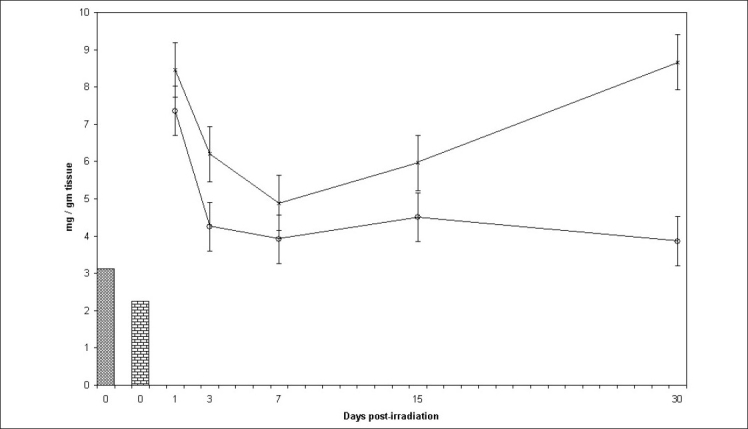
Cholesterol levels in mice testis at various post treatment days Variations in cholesterol level (mg/g tissue)±SD in testis of Swiss mice at various post treatment days, with and without supplementation of *Spinacia oleracea* extract (SE). Normal (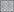) SE (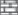), IR (––×––) and SE+IR (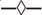)

Highly augmented levels of cholesterol in the control group at all post irradiation intervals declined by administration of SE by approximately 34.97%, 58.46%, 31.7%, 46.65% and 153.84% on day 1, 3, 7, 15 and 30 days post irradiation respectively (fig. 4). It seems that the available antioxidants in the spinach extract were able to cope up with the radiation induced oxidative stress to an extent. This might have been due to synergistic effects of the antioxidant constituents present. Difference between mean values of all four groups of cholesterol was significant (F=16.644, p<0.000).

## DISCUSSION

Results obtained from this study indicate that the spinach extract may act as radioprotective agent and render protection against radiation induced oxidative stress. Oxidative stress refers to the cytotoxic consequence of reactive oxygen by products: superoxide anions and hydroxyl radicals, which are generated as metabolites of normal and aberrant metabolic processes that utilize molecular oxygen^16^. Oxidative stress leads to lipid peroxidation, protein and carbohydrate oxidation and metabolic disorders^17^–^19^. The peroxyl radical formed through lipid peroxidation attack protein membrane and enzymes and reinitiates lipid peroxidation. The preservation of cellular membrane integrity depends on protection or repair mechanisms capable of neutralizing oxidative reactions.

The presence of antioxidants in the plants suppresses the formation of free lipid radical and thus prevents the formation of endoperoxidation. Spinach contains a very effective natural antioxidant system (NAO). The potential physiological role of NAO as an antioxidant was shown in several *in vitro* and *in vivo* systems^7^,^20^–^21^. The observed beneficial effects of supplemented vegetable intake may be contributed by the carotenoids; folate and Vitamin C. Currently knowledge on the bioavailability of these compounds from vegetables is limited. Van het Hoff *et al*,^22^ reported that the β-carotene supplemented meal increases the plasma concentration of β-carotene effectively. All vegetable meals increased the plasma concentration of lutein and Vitamin C significantly. A significant increase in plasma folate concentration was found only after consumption of the spinach-supplemented meal, which provided the highest level of folate. Disruption of the spinach matrix increased the plasma responses to both lutein and folate, whereas it did not affect the response to β-carotene. Guil *et al*,^23^ studied the nutritional (ascorbic acid, dehydroascorbic acid and carotenes), anti-nutritional and toxic components (oxalic acid, nitrate and uric acid) determined in sixteen popular species of East Spain.

Seifter and Collaborators have indicated the role of β-carotene and vitamin A in radiation protection due to their antioxidant properties^24^. Mill^25^ also observed a beneficial influence of β-carotene on the condition of patients undergoing radiotherapy. Cozzi *et al*,^26^ suggested that ascorbic acid and β-carotene are effective in reducing hydrogen peroxide induced sister chromatid exchange. They concluded that both β-carotene and vitamin A act as scavengers of endogenous and hydrogen peroxide induced reactive oxygen species. There is a growing body of evidence regarding the beneficial properties of β-carotene in human and animal diet^27^,^28^. Based on NMR spectroscopy, the major active components of NAO have been identified by Bergman *et al*,^29^ as glucuronic acid derivatives of flavonoids, trans and cis isomers of *p*-coumaric acid and *meso*-tartarate derivatives of coumaric acid, and uridine. alpha-lipoic acid (another glutathione-elevating agent) treatment for 28 days lowered lipid peroxidation among children chronically exposed to low doses of radiation in the area contaminated by the Chernobyl nuclear accident^30^.

The decrease of protein noted may be due to its lyses, by X-irradiation or may be at the synthesis level, or by the inhibition of release of synthesized polypeptides from polysomes^31^. Increased protein concentration recorded in our study at day 30 post exposure in SE supplemented irradiated mice is a beneficial effect. This proves an improvement in ribosomal activities, which enhance the protein synthesis, can be treated as antiradiation effect. Increase in protein concentration at day 30 post irradiation may also be due to the elimination of most of the degenerated cells from the tissue and thus the testicular weight loss or it may be due to the elimination of the most of the degenerated cells from the tissue and thus the testicular weight loss or it may be due to the increased demand of proteins in repair process as recovery is evident at day 30 post irradiation^32^.

Grant^33^ suggested that protection of protein is due to the hydrogen atom donation by the protector. Zhang and Omaye^34^ reported that high concentration of ß-carotene products produces more protein oxidation in the presence of high O_2_ tension by proxidant mechanism.

Initial increase in glycogen concentration suggested the increased energy requirement of degenerating and aberrant spermatogenic cell population. Similar results have been obtained by Gupta and Bawa^35^ who studied the effect of radiation on enzymes of carbohydrate metabolism. They observed that testicular hexokinase is highly sensitive to radiation damage. The reduced hexokinase activity seems to be related to those parts of testis (spermatocytes and spermatids), which depend on glucose for their functioning. Radiation induced atrophic testis is rich in glycogen content because of inhibition of glucose-6-phosphate as a phosphorylase may explain the high levels of polysaccharides although the possibility of enhanced glycogenolysis is due to activation of glycogen synthetase has also been suggested. Changamma and Redanna^36^ however suggested that the decrease in glycogen content could also be due to increased glycogenolysis. The presence of glycogen in the testis has been documented by Nicander^37^ who postulated its presence in the sertoli cells and germinal tissue indicating that it serves as an energy substrate for the developing spermatids.

The lowered concentration of cholesterol might be due to the higher activity of steroid synthesis, which is corresponding with the higher counts of spermatogenic cell population in the drug-exposed group. Irradiated animals exhibited an initial decline in cholesterol concentration in testis upto 7-day post irradiation that increased upto 30-post irradiation. Maini^38^ also observed the decrease in cholesterol concentration of the testis after whole body exposure of mice to 5 Gy gamma radiations. The increase in levels of cholesterol may be attributed to its decreased utilization for steroidogenesis, which may be due to pituitary inhibition or a direct inhibitory action of the target tissue^39^. Cholesterol present in sertoli cells, spermatogonia and spermatocytes. Impaired spermatogenesis results in a marked increase in cholesterol content regardless of the functional status of interstitial cells. It has also been suggested that the lipid fraction tends to increase when the testicular spermatozoal population depletes^40^.

In our laboratory Verma *et al*,^10^,^41^ has also reported the radioprotective efficacy of spinach against radiation induced oxidative stress in brain of male Swiss mice in terms of improved learning, lower LPO levels, improved protein synthesis. The reduction in the amount of TBARS equivalents, cholesterol and glycogen and increase in protein synthesis in SE administration irradiated animals suggests that SE may scavenge the free radicals during the radiation exposure, thus rendering protection against radiation induced oxidative stress. The result of present investigation supports the postulate that increased ROS induced by radiation exposure may be involved in some of the aversive effects of stress.

The antioxidative mechanism of β-carotene has been suggested to be singlet oxygen quenching. We used plant leaf extract, which aside from having high concentration of β-carotene content other constituents common to green plants the possible synergistic effect and possible beneficial potency of the plant nutrient obscure and warrants further research. The protection afforded with SE in biochemical activity of testis in the present study may prove to be beneficial for the clinical use of such dietary compound as radio protector.
